# Precocious Puberty: Types, Pathogenesis and Updated Management

**DOI:** 10.7759/cureus.47485

**Published:** 2023-10-22

**Authors:** Ahmed Alghamdi

**Affiliations:** 1 Pediatric Endocrinology, Faculty of Medicine, Al Baha University, Al Baha, SAU

**Keywords:** precocious puberty, mccune-albright syndrome, kiss1, mkrn3, gnrha, familial male-limited precocious puberty, dlk1, adrenal hyperplasia, aromatase inhibitors

## Abstract

Precocious puberty (PP) means the appearance of secondary sexual characters before the age of eight years in girls and nine years in boys. Puberty is indicated in girls by the enlargement of the breasts (thelarche) in girls and in boys by the enlargement of the testes in either volume or length (testicular volume = 4 mL, testicular length = 25 mm, or both). Two types of PP are recognized - namely central PP (CPP) and peripheral PP (PPP). This paper aims to describe the clinical findings and laboratory workup of PP and to illustrate the new trends in the management of precocious sexual maturation. Gonadotropin-releasing hormone (GnRH)-independent type (PPP) refers to the development of early pubertal maturation not related to the central activation of the hypothalamic-pituitary-gonadal (HPG) axis. It is classified into genetic or acquired disorders. The most common forms of congenital or genetic causes involve McCune-Albright syndrome (MAS), familial male-limited PP, and congenital adrenal hyperplasia. The acquired causes include exogenous exposure to androgens, functioning tumors or cysts, and the pseudo-PP of profound primary hypothyroidism. On the other hand, CPP is the most common and it is a gonadotropin-dependent form. It is due to premature maturation of the HPG axis. CPP may occur as genetic alterations, such as MKRN3, DLK1, or KISS1;as a part of mutations in the* *epigenetic factors that regulate the HPG axis, such as Lin28b and let-7; or as a part of syndromes, central lesions such as hypothalamic hamartoma, and others. A full, detailed history and physical examination should be taken. Furthermore, several investigations should be conducted for both types of PP, including the estimation of serum gonadotropins such as luteinizing and follicle-stimulating hormones and sex steroids, in addition to a radiographic workup and thyroid function tests. Treatment depends on the type of PP: Long-acting GnRHa, either intramuscularly or implanted, is the norm of care for CPP management, while in PPP, especially in congenital adrenal hyperplasia, the goal of management is to suppress adrenal androgen secretion by glucocorticoids. In addition, anastrozole and letrozole - third-generation aromatase inhibitors - are more potent for MAS.

## Introduction and background

Puberty is a primary period when sexual maturity and reproductive function are obtained and central somatic, psychological, and behavioral changes occur, indicating an adult phenotype [1]. Its neuroendocrine state, defined by complete stimulation of the hypothalamic-pituitary-gonadal (HPG) axis, comprises the following: (1) gonadotropin-releasing hormone (GnRH) from the hypothalamus; (2) gonadotropins from the pituitary [luteinizing hormone (LH) and follicle-stimulating hormone (FSH)]; and (3) gonadal steroids and peptides, which are induced by pituitary gonadotropins [2]. All of these steps are controlled by feedback mechanisms, either positive or negative [2].

The onset of pubertal changes occurs at two to 2.5 standard deviations (SD) below the mean age of onset of puberty (the age of eight to 13 years in girls and nine to 14 years in boys), depending on multifactorial elements such as genetic, environmental, metabolic, ethnic, geographic, nutritional, and economic factors, which are controlled by complex regulatory pathways [3].

Using Tanner staging, the definition of puberty is indicated in girls by the enlargement of the breasts (thelarche) and in boys by the enlargement of the testes, either in volume or in length (testicular volume = 4 mL, testicular length = 25 mm, or both); however, this definition continues to be subjective and arbitrary [4-6]. These changes are fundamental for the clinical diagnosis of pubertal pathology; thus, if they occur before the age of eight years in girls or nine years in boys, associated with linear growth and the acceleration of bone age [5, 6], then this is considered precocious puberty (PP). Therefore, the characteristic definition of precocious sexual maturation is the appearance of secondary sexual characteristics before the age of eight years in girls and nine years in boys [7]. By contrast, delayed puberty is described as the absence of somatic signs and changes related to pubertal development at an age two standard deviations above the mean (around the age of 13 years for both girls and boys) [8].

The aim of this paper is to define the PP, differentiate I between other conditions simulating PP, differentiate between peripheral and central types, identify syndromes involved in PP, describe the clinical findings, laboratory investigations, genetic basis, diagnosis methods, hormonal assessment and using GnRH test and cutoff points, radiological findings, thyroid functions, and other workups of PP. Furthermore, it also aims to illustrate the new trends in the management of PP such as using aromatase inhibitors, and implants.

## Review

While some researchers have proposed that thelarche currently occurs earlier than it was reported to in the 1960s, the age of menarche, especially in industrialized countries, has remained comparatively steady, following a period of gradual decay until the twentieth century [9]. Consequently, the period between thelarche and menarche appears to have grown [10]. The age border for precocious sexual maturation in girls has been reestimated and reidentified in the USA following epidemiological data obtained from several studies. These studies have revealed that the signs of puberty are developed at the age of six years in Black girls and seven years in White girls, especially in those of African-American descent [11, 12]. However, many debates about the reliability of these studies have been raised because they have estimated the truth through inspection only. Furthermore, a reduction of the age threshold for estimating precocious puberty may lead to some girls being declared to have dedicated fast advanced PP, potentially leading to the misdiagnosis of probably curable underlying factors [13].

In general, most relevant studies have revealed that precocious sexual maturation is more common in girls than in boys. In the USA, the estimated incidence was one case per 5000-10,000 girls, with 15-20 affected girls for every boy [12]. In Denmark, the estimated incidence was 0.2% for girls and less than 0.05% for boys [13]. In Spain, the estimated incidence was 37 cases per 100,000 girls, compared with 0.46 per 100,000 boys [14]. In South Korea, the estimated rate was 55.9 cases per 100,000 girls, compared with 1.7 per 100,000 boys [15].

Two types of precocious sexual maturation are recognized - namely central PP (CPP) and peripheral PP (PPP) [16]. Primarily, it is crucial to differentiate between CPP and other cases that simulate CPP, such as isolated premature adrenarche and isolated premature thelarche (IPT) [17, 18]. Premature adrenarche is described as pubarche, which denotes the presence of axillary and pubic hair, acne, and apocrine body odor [19]. Premature adrenarche is caused by an increase in the level of adrenal androgen-independent of the normal HPG axis [18, 19]. It is diagnosed by exclusion after declaring other pathologic conditions associated with androgen excess. It usually appears before the age of eight years in girls and nine years in boys [20]. While premature adrenarchy has a good outcome and is considered a benign condition, caution should be considered and the child should be monitored periodically for the early detection of other pubertal signs [21]. On the other hand, IPT is defined as the appearance of breast tissue alone without other signs suggestive of puberty, such as augmented growth velocity, advanced skeletal maturation, and rapid development of the breasts. The outcome of IPT is usually benign, self-limited, and regressive over time from a few months to years), and it rarely progresses to CPP [22].

Peripheral precocious puberty

**Figure 1:**
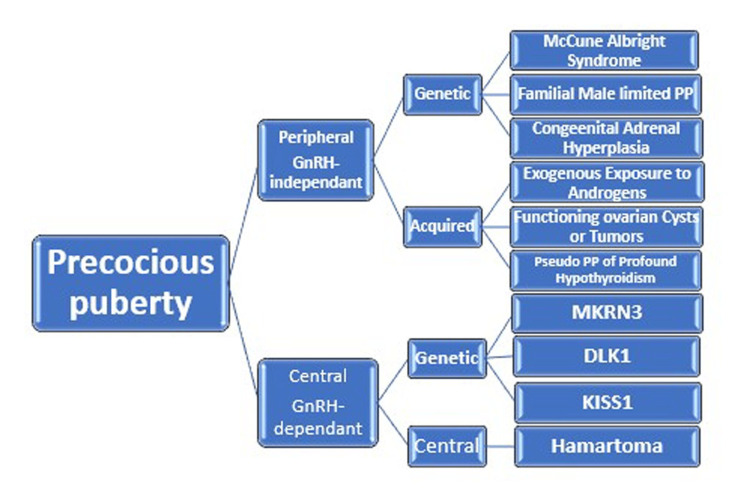
This algorithm illustrates the types and etiology of precocious puberty.

PPP, the so-called GnRH-independent type, is the presence of early pubertal maturation not related to the central activation of the hypothalamic-pituitary-gonadal (HPG) axis. The etiology of PPP is classified into genetic or acquired disorders. The most common forms of congenital or genetic causes include familial male-limited PP (FMPP), McCune-Albright syndrome (MAS), and congenital adrenal hyperplasia (CAH) [23]. By contrast, the acquired causes include exogenous exposure to androgens, functioning tumors or cysts, and the pseudo-PP of profound primary hypothyroidism [24]. Two types of PPP have been described - namely the acquired and congenital forms (Figure 1).

Acquired Peripheral Precocious Puberty

Acquired PPP may originate from exposure to either exogenous or excess endogenous sex steroids from, for example, sex steroid-secreting tumors in the gonads (e.g., testicular Leydig cell tumors [25]) ovarian granulosa cell tumors [24], or adrenal tumors (e.g., adrenocortical tumors [26]). In addition, PPP may occur as a result of a germline mutation in the p53 tumor-suppression gene, resulting in the formation of Li-Fraumeni syndrome [27]. Said syndrome is characterized by excess production of human chorionic gonadotropin (hCG) from germ cell tumors, which has a direct effect on testicular Leydig cells through its affinity to bind with the luteinizing hormone (LH) receptor [28]. This leads to the excessive production of testosterone, which causes PP in boys.

Numerous possible sources of exogenous exposure to estrogens or androgens have been reported. They include the inadvertent ingestion of anabolic steroids or oral contraceptives; skin-to-skin transmission of topical androgen preparations, such as testosterone gel, from parents to their children [29]; systemic absorption from hair products containing placental-related extracts or estrogen [30]; and the topical use of lavender oil or tea tree oil, which has been described as a potential cause of gynecomastia in the prepubertal period [31].

A form of PPP that is poorly understood is pubic hair of infancy. It is defined as the development of isolated pubic hair in infants of both male and female sexes aged four to eight months [9, 10]. Physical examinations are normal among affected infants; however, the distribution of hair is characteristic of this condition, as it is restricted only to the scrotum in boys and the mons in girls. This is explained by one of two postulated mechanisms - either an increased concentration of urinary sex steroids during the mini puberty of infancy or premature adrenarchy, as evidenced by elevated dehydroepiandrosterone sulfate levels estimated by mass spectrometry [11]. However, the condition seems benign, and long-term follow-up is recommended.

McCune-Albright Syndrome

MAS is characterized by PP, café-au-lait skin pigmentation, and fibrous dysplasia of the bone. Some patients present with PP associated with other endocrinopathies, such as hyperthyroidism, Cushing syndrome, or other atypical features. MAS is produced by a somatic-activating mutation in the guanine nucleotide-binding protein alpha-stimulating activity polypeptide (GNAS), which is involved in controlling the production of various hormones responsible for regulating the activity of endocrine glands, such as the pituitary, thyroid gland, gonads, and adrenal glands. This gene encodes the stimulatory subunit Gs, which is a part of the intracellular signaling cascade present in all endocrine cells as well as other tissues. The mutation occurs in mosaic form, and the syndrome itself is illustrated by a highly heterogeneous variety of manifestations, severity levels, and outcomes [32].

Sudden painless vaginal bleeding in girls, accompanied by subtle and acute breast enlargement, is caused by the activation of G-protein signaling in the ovaries. This has consequences for autonomous function with the gradually progressive development of unilateral ovarian estrogen-secreting cysts. With the involution of cysts, the estrogen level decreases, and vaginal bleeding starts. While fibrous dysplasia in young children is often asymptomatic, the diagnosis of MAS can be established by the presence of the increased uptake of radioisotopes in a bone scan. However, efforts to perform a molecular analysis of GNAS in peripheral blood to prove suspected MAS are often ineffective [33].

Moreover, the clinical presentation of PP-related MAS is highly variable. Many girls have widened intervals of dormancy, while others encounter repeated attacks of vaginal bleeding. Some parents of girls with MAS also only experience breast development. Because of the variable clinical presentation, course, and outcome, an observation period is always proposed before starting therapy. The main goal in the management of PP-related MAS should be directed toward the psychological impact of vaginal bleeding and the patient’s height due to premature closure of epiphyses because of estrogen exposure [34].

Central precocious puberty

I*ntroduction to CPP*

CPP occurs as a result of premature maturation of the HPG axis, and it is considered the most common form of PP as it accounts for approximately 80% of cases; accordingly, it is gonadotropin-dependent [35]. By contrast, PPP is caused by excessive production of sex hormones either from an exogenous or tumoral source or due to a genetic disease that is considered gonadotropin-independent [8]. 

Younger age and male gender are considered the primary risk factors for developing CPP [7]. The most common form is idiopathic CPP, which accounts for approximately 75-90% of cases in girls and 25-60% in boys [16]. However, the causes for both girls and boys are similar. The most commonly involved brain disorders are meningomyelocele, hydrocephalus, encephalitis, hamartoma in the hypothalamus, hypoxic-ischemic encephalopathy of the neonates, neurofibromatosis type 1 [36], which are significantly more common in boys than girls [37-39]. 

Types of genetic CPP 

MKRN3: Mutations in the MKRN3 gene in familial CPP began to be documented in 2013, and their detection sparked a state of evolution in understanding the genetic basis of CPP [40]. MKRN3 encodes an intracellular protein named makorin RING finger protein 3, which is involved in cellular processes such as protein-protein interactions, protein degradation, protein stability, and protein quality control. Several studies on rodents and primates have found the highest levels of MKRN3 in the key hypothalamic areas, with the highest point during the early period of life before gradually decreasing over time, especially prior to the pubertal period [41, 42].

Recently, MKRN3 was demonstrated to block KISS1 and TAC3 promoter activity, thus acting as a blocking agent for the hypothalamic pathway for neuronal GnRH. Besides KNDy and kisspeptin neuron goals, MKRN3 was found to repress the promoter of GNRH1 by directly affecting the methyl-CpG-binding protein [43].

In MKRN3 mutations, no additional clinical signs have been reported elsewhere in CPP, and patients are usually referred to clinical advice for the appearance of primary signs of PP, such as testicular enlargement in boys and early thelarche in girls [42]. The incidence of MKRN3-CPP exhibits female predominance, with a female-to-male ratio of 6:1. Furthermore, girls with MKRN3 have the tendency to develop the disease earlier than boys; some reports indicate that PP may start at the age of six years in girls, compared with 8.5 years in boys [43]. Additionally, FSH levels were found to be higher in girls than boys [43]. On the other hand, boys with MKRN3 mutations may display puberty onset at an age close to normal puberty, and the diagnosis of PP would be made because of the presence of a strong positive family history of CPP, especially in first-degree relatives [44].

DLK1: Another form of genetic mutation was discovered in families that exhibited rearrangements of genomes within chromosome 14, which harbors the DLK1 gene [45]. During family counseling and the formation of the family pedigree, the maternal imprinting was confirmed, and individuals demonstrated CPP merely when the mutation was inherited from their fathers. Patients with the mutant DLK1 gene exhibited advanced bone age, and the disease started early at the ages of 4.5 to six years [46]. The diagnosis of CPP was documented by both elevated levels of basal and stimulated LH. Girls responded to treatment with GnRH analogs and reached a height close to that of normal individuals.

KISS1: Rare, isolated cases of mutant KISS1 genes have been explained in CPP [47]. KISS1 [and KISS1R a (GPR54)] is a G protein-coupled receptor [47] that plays a crucial role in regulating reproduction, metabolic function, and pubertal maturation [48-50]. It encodes the neuropeptide kisspeptin or metastin, the natural ligand of the kisspeptin receptor.

The mutation is inherited as variable expressivity or polygenism from unaffected maternal factors, such as the mother and grandmother [51]. The signs of pubertal development in male patients appear early at the age of 17 months. To date, no molecular credentials have been afforded for these variants; therefore, it is difficult to prove an underlying relationship between the KISS1 mutation and the development of CPP [52].

Epigenetic factors: Beyond protein-coding genes, epigenetic factors are also known to regulate the HPG axis, such as Lin28b and Let 7 [53]. Lin28 is a microRNA repressor, while Let 7 is a differentiating microRNA that is mutually controlled by Lin28b [54]. The expression level of Lin28b decreases in the first stages of pubertal activation, while the levels of Let 7 increase [54].

CPP as a Part of Syndromes

Pallister-Hall syndrome is characterized by syndactyly, polydactyly, neurological signs, midline defects, and the development of CPP. The diagnosis can be proven by the presence of the GL13 mutation in molecular genetic studies. Most of this mutation is inherited as autosomal dominant, while approximately 25% may be de novo [55].

Cowden and Cowden-like syndromes are caused by mutations in the PTEN, KLLN, and SDHB-D genes [56]. These syndromes are described by the associations of multiple hamartomas, which may give rise to CPP if they include the infundibulum or the hypothalamus.

Temple syndrome is a maternally imprinted disease that results from maternal uniparental disomy or paternal deletion of chromosome 14. It is characterized by growth retardation, short stature, small hands, truncal hypotonia, facial dysmorphism, and the development of CPP [57]. The CPP may be a part of this syndrome or appear as an isolated form related to the DLK1 mutation (Figure 1).

These changes are fundamental for the clinical diagnosis of pubertal pathology; thus, if they occur before the age of eight years in girls or nine years in boys, associated with linear growth and the acceleration of bone age [5, 6], then this is considered precocious puberty (PP). Therefore, the characteristic definition of precocious sexual maturation is the appearance of secondary sexual characteristics before the age of eight years in girls and nine years in boys [7]. By contrast, delayed puberty is described as the absence of somatic signs and changes related to pubertal development at an age two standard deviations above the mean (around the age of 13 years for both girls and boys) [8].

The aim of this paper is to define the PP, differentiate between other conditions simulating PP, differentiate between peripheral and central types, identify syndromes involved in PP, describe the clinical findings, laboratory investigations, genetic basis, diagnosis methods, hormonal assessment, and using GnRH test and cutoff points, radiological findings, thyroid functions, and other workups of PP. Furthermore, it also aims to illustrate the new trends in the management of PP such as using aromatase inhibitors, and implants.

Prader-Willi syndrome (PWS) results from the imprinted locus on chromosome 15q11, in which MKRN3 is included, among other genes [58]. PWS is characterized by morbid obesity, dysmorphisms, and intellectual and behavioral problems [59]. CPP may be a part of this syndrome or appear as an isolated form related to MKRN3 mutation.

Neurofibromatosis type 1 (NF-1) results from a mutant form of the NF1 gene, which is inherited as an autosomal dominant trait. The associated CPP was found in approximately 2.5% of NF1 gene-affected patients [60].

Tuberous sclerosis (Bourneville disease) results from a mutation in the TSC1 and TSC2 genes. This mutation gives rise to multisystemic disorders and hamartomas, which may manifest as CPP [61]. 

Williams-Beuren syndrome results from a deletion in chromosome 7q11.23. It is characterized by cognitive and developmental anomalies, cardiac abnormalities, facial dysmorphisms, and tumors with associated early pubertal development, especially in cases with central nervous system (CNS) tumors [62].

Other syndromes were also reported to have an association with CPP, such as Silver-Russell syndrome (SRS) [63]. The prevalence of CPP in these patients is unknown. Cohen syndrome [64] and Stankiewicz-Slider syndrome [65] are caused by deletions of chromosome 17q24 or PSMD12 mutations, while mucopolysaccharidosis type IIIA, a metabolic disorder related to SGSH mutations, gives rise to severe neurological and sensorineural features [66].

CNS lesions: The pathogenesis of hypothalamic hamartomas in activating GnRH neurons is questioned. It may activate surrounding glial cells (and secondarily GnRH) through TGFα [67] or provide indirect evidence through the presence of GnRH immunoreactivity within the tumor that indicates excess GnRH production [68].

Gliomatous cells have been reported to express the GnRH receptor [69]. Other lesions, including pineal cysts, hydrocephalus, infiltrative lesions, meningomyelocele, infectious diseases, and encephalopathies, were reported to be associated with CPP [69].

Social stressors and nutritional imbalances: Adoption is one of the most widely recognized risk factors for CPP [15]. However, the causal relationship between adoption and the mechanism of evolving CPP is still unclear [70]. 

Workup of precocious puberty

A full, detailed history must be taken from patients suspected of having PP. This includes the patient’s age, age of onset of physical changes and rate of progression, state of sex steroids (either external or internal exposure), drug use or accidental ingestion of contraceptive pills, skin contact with absorbable testosterone gel, and exposure to estrogens or androgen-containing substances [71]. Symptoms associated with the CNS include headache, visual disturbances, polydipsia, polyuria, and behavioral/mood changes, history of brain trauma, CNS infection, and neonatal history/family history. Family history includes the age of pubertal onset in both parents, siblings, and other family members (i.e., voice change, age of first menarche, voice breaking, and growth spurt).

Physical examination depends on the application of Tanner staging to assess pubertal changes, such as breast enlargement in girls, penile development and measurement of testicular volume in boys, and the presence of pubic hair in both sexes. In addition, it is used to assess anthropometric measurements and estimate growth velocity [72].

The primary sign for assuming the start of puberty is thelarche in girls and an enlargement of the testes by 4 mL in boys. The orchidometer should be used to measure the volume of testis differentiating between bilateral and unilateral testicular enlargement and examining for the presence of testicular masses. A physical examination should be performed to differentiate signs of PP from other simulated findings, such as lipomastia (i.e., the accumulation of fatty tissue in the breast, which is seen in obese and overweight girls). Additionally, abnormal findings in the skin, such as the presence of café-au-lait spots, suggest an association with neurofibromatosis type 1 or MAS [73].

Furthermore, heights should be mapped periodically on a growth chart, as an increase in height of one full percentile space of more establishes a diagnosis of PP [74, 75]. Furthermore, the height should be assessed against the mid-parental height, using the following formulas: for boys - ([mother’s height cm + 13 cm] + father’s height cm) / 2; and for girls - ([father’s height cm − 13 cm] + mother’s height cm) / 2 [72, 76].

Moreover, clinical presentation, the order of pubertal changes, and the rate of progression may differ between PPP and CPP. The differences may include the onset being either sudden or gradual, intermittent, and involving changes related to estrogens, androgens, or both [77, 78].

In general, some features may suggest the type of PP. For example, a testicular volume less than 4 mL with pubic hair development and penile growth suggests a diagnosis of PPP, while increased testicular volume greater than 4 mL in association with other signs of puberty suggest CPP [9], except in familial male-limited precocious puberty (FMPP or testotoxicosis) and hCG-secreting germ cell tumors, in which mild testicular enlargement is present. FMPP, or testotoxicosis, is a part of PPP due to a mutation of the LH receptor, resulting in overactivation and increased Leydig cell secretion of testosterone [75, 76, 79, 80].

Furthermore, adrenal tumors can exhibit signs of virilization related to androgen excesses, such as pubic hair and clitoromegaly in girls. This is in addition to signs related to excess glucocorticoids, such as a rapid gain in body weight, facial plethora, a round (moon) face, the appearance of striae, hirsutism, and hypertension and its related systemic changes [77].

Other presenting signs may suggest specific causes; for example, vaginal bleeding with sudden onset associated with minimal or no breast development may suggest MAS, which is caused by a mutation in the α-subunit of the G-protein [78, 79]. In girls affected by MAS, PPP is produced by excessive estrogen secretion from the accompanying functioning ovarian cysts [80]. The cause of vaginal bleeding is the cessation of estrogen following the involution of a cyst [81, 82]. Other findings, alongside the appearance of precocious sexual maturation, recommend a diagnosis of MAS, including café-au-lait spots and polyostotic fibrous dysplasia of the bone [82].

Hormonal Assessment

The first step in the laboratory work for PP is the estimation of serum gonadotropins and sex steroids. Lowered secretion of FSH in association with high levels of sex steroids suggests a diagnosis of PPP [6, 22].

LH should be sampled in the early morning using a detection kit of 0.1 IU/L [6]. Several reports have assessed the basal LH to exclude CPP, with cutoff points varying from 0.1 to 1 IU/L [82-85]. The basal LH sensitivity for the mere diagnosis of CPP varies between 50% and 100%, with a range of specificity of 64-100% [7].

In some instances, caution should be employed when interpreting gonadotropin concentrations, especially in children below the age of two years. This is because elevated levels of both LH and FSH may be considered physiological owing to the presence of mini puberty at this age [4, 6].

To distinguish CPP from thelarche, clinical monitoring of pubertal progression and growth should be conducted [85]. When CPP is doubted in the association of nonconfirmatory ambiguous basal LH, a GnRH stimulation test is required. This involves injecting a short-acting GnRH as gonadorelin at a dose of 100 μg, and LH should be collected in one blood sample 30-40 min following GnRH injection [86]. Alternatively, a long-acting GnRH agonist (GnRHa), such as leuprorelin in a dose of 3.75 mg, can be used, with the estimation of LH in one blood sample conducted at 30-180 min.

To diagnose active puberty following either GnRH or GnRHa stimulation tests, the cutoff point of LH should be more than 5 IU/L [87, 88]; however, further cutoff points, varying from 4 to 8 IU/L, have also been proposed [7]. Another peak of the LH-to-FSH ratio of 0.6-1.0 following a GnRHa-stimulated test was introduced as a sign of active puberty; however, its sensitivity and specificity were low compared with LH alone [89]. The hypersensitivity reaction and high cost are considered disadvantages of GnRHa simulation tests.

On the other hand, in boys, the concentration of testosterone in the early morning is a useful indicator of the presence of sexual precocity [20]. However, in girls, low serum estradiol levels do not rule out the PP [90]. However, high concentrations of estradiol in the association of decreased gonadotropins strongly indicate PPP [41]. 

In addition, thyroid function should be evaluated to rule out the diagnosis of pseudo-PP related to long-standing hypothyroidism [91]. Furthermore, insulin-like growth factor-1 (IGF-1) and its correlation with insulin levels should be studied, as IGF-1 exhibits an increased level in early puberty, which assists in the diagnosis of CPP [92].

**Figure 2:**
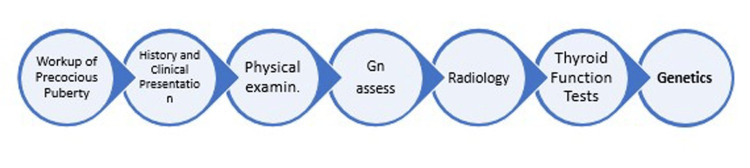
This figure illustrates the workup of precocious puberty either central or peripheral.

Moreover, bone age should be assessed through a left hand and wrist radiograph using either the Tanner-Whitehouse 3 (TW3) or Greulich and Pyle atlas method [76, 93, 94]. Today, updated automated estimation systems using artificial intelligence are used [95]. Advanced bone age usually occurs in cases of precocious maturation; a significantly advanced maturation is considered when the advancement goes beyond one to standard deviations in chronological age (Figure 2) [96].

Treatment

CPP

Long-acting GnRHa, either intramuscularly or implanted, is the norm of care for the management of CPP. The action of GnRHa depends on persistently high GnRH concentrations, which result in the suppression of the HPG axis, thereby stopping gonadotropin secretion [97]. Hormonal treatment results in steadying pubertal progression, a regressing growth velocity, and a reduction in bone age advancement [98].

The most reported side effects of GnRHa are hot flushes, headaches, and mild reactions at injection sites [99]. Rarely, intramuscular injections or implants may result in the development of a sterile abscess, which has led to negative feedback on the treatment’s efficacy [16, 100-102].

In girls, vaginal bleeding may occur following the first injection owing to the temporary increase in the level of estradiol [7]. Some studies have also reported that patients with GnRHa gained weight [103, 104].

An implant is enclosed into the upper inner arm using local anesthesia, resulting in rapid and sincere HPG axis suppression for one year [105]. A concern regarding the use of these implants is the risk of the device cracking during extraction [106]. Monitoring of the treatment is regulated by clinical factors, such as Tanner stage, skeletal maturation, and linear growth.

Failure of treatment is indicated by persistent testicular or breast development, advancement of the bone age, and high growth velocity [107]. In such cases, modification of the dosage form, either increasing the dose or using intervals, should be kept in mind [108]. LH levels stimulation using GnRH, either free GnRHa or aqueous GnRHa contained in depot form, can be used to estimate treatment [109, 110-112]. The decrease of LH secretion to less than 2.5-4.5 IU/L is a sufficient goal in patients on monthly GnRHa therapy [113-116].

PPP

In CAH, the goal of management is to suppress adrenal androgen secretion with glucocorticoids. In MAS, girls who exhibit promptly advancing puberty, accelerated growth, repeated menses, and advanced bone age may show improvement with this treatment [117]. In addition, aromatase inhibitors inhibit the conversion of androgens to estrogens and are useful in the management of MAS in girls through binding to the cytochrome P450 [118, 119].

Today, anastrozole and letrozole - third-generation aromatase inhibitors - are more potent than previous generations of agents, with letrozole in particular demonstrating greater effectiveness in the treatment of MAS. In addition, fulvestrant and tamoxifen are considered adjuvant therapies or second-line treatments [120]. The treatment protocol includes letrozole either in the form of a single daily dose of 2.5 mg for the whole treatment period or as gradually rising doses, starting with 0.5 mg/m2/day for seven days, which increases nearly every week by 0.5 mg/ m2/day up to 1.5 mg/m2/day. A dose of 2 mg/m2/day is considered if any worsening occurs [121]. Bisphosphonate compounds, such as zoledronate and pamidronate, are recommended for patients with persistent bone pain [122]. Surgery should be avoided owing to the bilateral nature of the disease [123].

**Figure 3:**
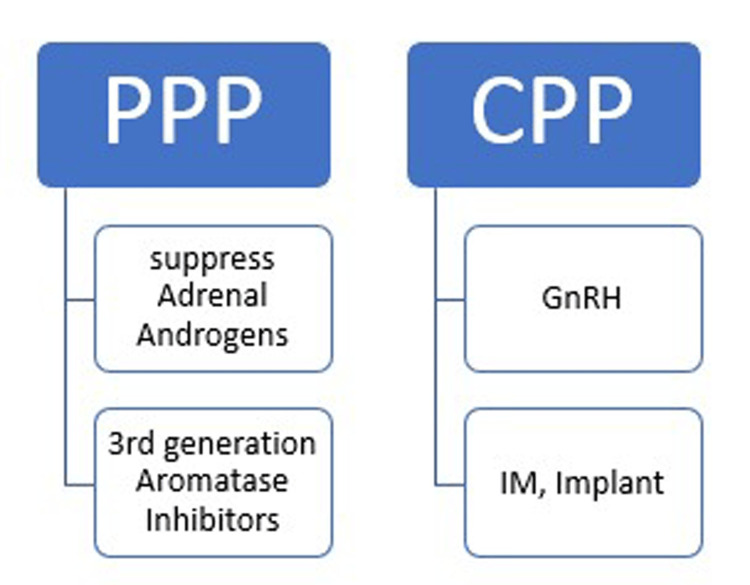
This graph illustrates the broad outlines of the treatment of peripheral precocious puberty (PPP) and central peripheral precocious puberty (CPP). IM: intramuscular

Lastly, the management of FMPP involves an antiandrogen in combination with a third-generation aromatase inhibitor. The most widely used is spironolactone, with a starting dose of 5.7 mg/kg/day and increasing up to 500 mg/day in combination with anastrozole (1 mg/day) [119]. Ketoconazole and cyproterone acetate have also been advocated [120]. For sex steroid-secreting tumors, surgical resection is considered the first-line therapy except for in the case of functioning follicular ovarian cysts, which regress over time (Figure 3) [121]. 

## Conclusions

PPP involves FMPP, MAS, and CAH. CPP is the most common and gonadotropin-dependent form and occurs due to premature maturation of the HPG axis. CPP may incorporate genetic alterations, such as MKRN3, DLK1, and KISS1; mutations in the epigenetic factors that regulate the HPG axis, such as Lin28b and Let 7; syndromes; central lesions such as hypothalamic hamartoma; and others. A full, detailed history and thorough physical examination must be conducted.

Additionally, several investigations should be obtained for both types of PP, including the measurement of serum gonadotropins and sex steroids, in addition to a radiographic workup and thyroid function tests. Treatment for CPP includes long-acting GnRHa, either injected intramuscularly or implanted, while in PPP - especially CAH - the main goal is the suppression of adrenal androgen secretion by glucocorticoids. Lastly, the third-generation aromatase inhibitors anastrozole and letrozole are more potent for MAS.

## References

[1] Shahab M, Mastronardi C, Seminara SB, Crowley WF, Ojeda SR, Plant TM (2005). Increased hypothalamic GPR54 signaling: a potential mechanism for initiation of puberty in primates. Proc Natl Acad Sci U S A.

[2] Bianco SD (2012). A potential mechanism for the sexual dimorphism in the onset of puberty and incidence of idiopathic central precocious puberty in children: sex-specific kisspeptin as an integrator of puberty signals. Front Endocrinol (Lausanne).

[3] Livadas S, Chrousos GP (2019). Molecular and environmental mechanisms regulating puberty initiation: an integrated approach. Front Endocrinol (Lausanne).

[4] Lanciotti L, Cofini M, Leonardi A, Penta L, Esposito S (2018). Up-to-date review about minipuberty and overview on hypothalamic-pituitary-gonadal axis activation in fetal and neonatal life. Front Endocrinol (Lausanne).

[5] Marshall WA, Tanner JM (1970). Variations in the pattern of pubertal changes in boys. Arch Dis Child.

[6] Kim SJ, Kim JH, Hong YH (2023). 2022 Clinical practice guidelines for central precocious puberty of Korean children and adolescents. Ann Pediatr Endocrinol Metab.

[7] Latronico AC, Brito VN, Carel JC (2016). Causes, diagnosis, and treatment of central precocious puberty. Lancet Diabetes Endocrinol.

[8] Cabrera SM, DiMeglio LA, Eugster EA (2013). Incidence and characteristics of pseudoprecocious puberty because of severe primary hypothyroidism. J Pediatr.

[9] Bradley SH, Lawrence N, Steele C, Mohamed Z (2020). Precocious puberty. BMJ.

[10] Sørensen K, Mouritsen A, Aksglaede L, Hagen CP, Mogensen SS, Juul A (2012). Recent secular trends in pubertal timing: implications for evaluation and diagnosis of precocious puberty. Horm Res Paediatr.

[11] Herman-Giddens ME, Slora EJ, Wasserman RC, Bourdony CJ, Bhapkar MV, Koch GG, Hasemeier CM (1997). Secondary sexual characteristics and menses in young girls seen in office practice: a study from the Pediatric Research in Office Settings network. Pediatrics.

[12] Kaplowitz PB, Oberfield SE (1999). Reexamination of the age limit for defining when puberty is precocious in girls in the United States: implications for evaluation and treatment. Drug and Therapeutics and Executive Committees of the Lawson Wilkins Pediatric Endocrine Society. Pediatrics.

[13] Teilmann G, Pedersen CB, Jensen TK, Skakkebaek NE, Juul A (2005). Prevalence and incidence of precocious pubertal development in Denmark: an epidemiologic study based on national registries. Pediatrics.

[14] Soriano-Guillén L, Corripio R, Labarta JI, Cañete R, Castro-Feijóo L, Espino R, Argente J (2010). Central precocious puberty in children living in Spain: incidence, prevalence, and influence of adoption and immigration. J Clin Endocrinol Metab.

[15] Kim SH, Huh K, Won S, Lee KW, Park MJ (2015). A significant increase in the incidence of central precocious puberty among korean girls from 2004 to 2010. PLoS One.

[16] Khokhar A, Mojica A (2018). Premature thelarche. Pediatr Ann.

[17] Novello L, Speiser PW (2018). Premature adrenarche. Pediatr Ann.

[18] Rieger E, Kofler R, Borkenstein M, Schwingshandl J, Soyer HP, Kerl H (1994). Melanotic macules following Blaschko's lines in McCune-Albright syndrome. Br J Dermatol.

[19] DeSalvo DJ, Mehra R, Vaidyanathan P, Kaplowitz PB (2013). In children with premature adrenarche, bone age advancement by 2 or more years is common and generally benign. J Pediatr Endocrinol Metab.

[20] Brito VN, Latronico AC, Arnhold IJ, Mendonça BB (2008). Update on the etiology, diagnosis and therapeutic management of sexual precocity. Arq Bras Endocrinol Metabol.

[21] Eugster EA (2009). Peripheral precocious puberty: causes and current management. Horm Res.

[22] Schultz KA, Sencer SF, Messinger Y, Neglia JP, Steiner ME (2005). Pediatric ovarian tumors: a review of 67 cases. Pediatr Blood Cancer.

[23] Mengel W, Knorr D (1983). Leydig cell tumours in childhood. Prog Pediatr Surg.

[24] Narasimhan KL, Samujh R, Bhansali A, Marwaha RK, Chowdhary SK, Radotra BD, Rao KL (2003). Adrenocortical tumors in childhood. Pediatr Surg Int.

[25] Stecher CW, Grønbaek K, Hasle H (2008). A novel splice mutation in the TP53 gene associated with Leydig cell tumor and primitive neuroectodermal tumor. Pediatr Blood Cancer.

[26] Shinoda J, Sakai N, Yano H, Hattori T, Ohkuma A, Sakaguchi H (2004). Prognostic factors and therapeutic problems of primary intracranial choriocarcinoma/germ-cell tumors with high levels of HCG. J Neurooncol.

[27] Kunz GJ, Klein KO, Clemons RD, Gottschalk ME, Jones KL (2004). Virilization of young children after topical androgen use by their parents. Pediatrics.

[28] Tiwary CM (1998). Premature sexual development in children following the use of estrogen- or placenta-containing hair products. Clin Pediatr (Phila).

[29] Henley DV, Lipson N, Korach KS, Bloch CA (2007). Prepubertal gynecomastia linked to lavender and tea tree oils. N Engl J Med.

[30] Nebesio TD, Eugster EA (2006). Pubic hair of infancy: endocrinopathy or enigma?. Pediatrics.

[31] Papadimitriou A, Beri D, Nicolaidou P (2006). Isolated scrotal hair in infancy. J Pediatr.

[32] Kaplowitz P, Soldin SJ (2007). Steroid profiles in serum by liquid chromatography-tandem mass spectrometry in infants with genital hair. J Pediatr Endocrinol Metab.

[33] Zacharin M (2007). The spectrum of McCune Albright syndrome. Pediatr Endocrinol Rev.

[34] Nabhan ZM, West KW, Eugster EA (2007). Oophorectomy in McCune-Albright syndrome: a case of mistaken identity. J Pediatr Surg.

[35] Wagoner HA, Steinmetz R, Bethin KE, Eugster EA, Pescovitz OH, Hannon TS (2007). GNAS mutation detection is related to disease severity in girls with McCune-Albright syndrome and precocious puberty. Pediatr Endocrinol Rev.

[36] Abreu AP, Kaiser UB (2016). Pubertal development and regulation. Lancet Diabetes Endocrinol.

[37] Partsch CJ, Sippell WG (2001). Pathogenesis and epidemiology of precocious puberty. Effects of exogenous oestrogens. Hum Reprod Update.

[38] Partsch CJ, Heger S, Sippell WG (2002). Management and outcome of central precocious puberty. Clin Endocrinol (Oxf).

[39] Cheuiche AV, da Silveira LG, de Paula LC, Lucena IR, Silveiro SP (2021). Diagnosis and management of precocious sexual maturation: an updated review. Eur J Pediatr.

[40] Abreu AP, Dauber A, Macedo DB (2013). Central precocious puberty caused by mutations in the imprinted gene MKRN3. N Engl J Med.

[41] Abreu AP, Toro CA, Song YB (2020). MKRN3 inhibits the reproductive axis through actions in kisspeptin-expressing neurons. J Clin Invest.

[42] Heras V, Sangiao-Alvarellos S, Manfredi-Lozano M (2019). Hypothalamic miR-30 regulates puberty onset via repression of the puberty-suppressing factor, Mkrn3. PLoS Biol.

[43] Li C, Lu W, Yang L (2020). MKRN3 regulates the epigenetic switch of mammalian puberty via ubiquitination of MBD3. Natl Sci Rev.

[44] Valadares LP, Meireles CG, De Toledo IP (2019). MKRN3 mutations in central precocious puberty: a systematic review and meta-analysis. J Endocr Soc.

[45] Maione L, Naulé L, Kaiser UB (2020). Makorin RING finger protein 3 and central precocious puberty. Curr Opin Endocr Metab Res.

[46] Dauber A, Cunha-Silva M, Macedo DB (2017). Paternally inherited DLK1 deletion associated with familial central precocious puberty. J Clin Endocrinol Metab.

[47] Silveira LG, Noel SD, Silveira-Neto AP (2010). Mutations of the KISS1 gene in disorders of puberty. J Clin Endocrinol Metab.

[48] Lee DK, Nguyen T, O'Neill GP (1999). Discovery of a receptor related to the galanin receptors. FEBS Letters.

[49] Roseweir AK, Millar RP (2009). The role of kisspeptin in the control of gonadotrophin secretion. Hum Reprod Update.

[50] Tolson KP, Garcia C, Yen S (2014). Impaired kisspeptin signaling decreases metabolism and promotes glucose intolerance and obesity. J Clin Invest.

[51] Song WJ, Mondal P, Wolfe A (2014). Glucagon regulates hepatic kisspeptin to impair insulin secretion. Cell Metab.

[52] Mazaheri A, Hashemipour M, Salehi M, Behnam M, Hovsepian S, Hassanzadeh A (2015). Mutation of kisspeptin 1 gene in children with precocious puberty in isfahan city. Int J Prev Med.

[53] Ong KK, Elks CE, Wills AK (2011). Associations between the pubertal timing-related variant in LIN28B and BMI vary across the life course. J Clin Endocrinol Metab.

[54] Sangiao-Alvarellos S, Manfredi-Lozano M, Ruiz-Pino F (2013). Changes in hypothalamic expression of the Lin28/let-7 system and related microRNAs during postnatal maturation and after experimental manipulations of puberty. Endocrinology.

[55] Démurger F, Ichkou A, Mougou-Zerelli S (2015). New insights into genotype-phenotype correlation for GLI3 mutations. Eur J Hum Genet.

[56] Mahdi H, Mester JL, Nizialek EA, Ngeow J, Michener C, Eng C (2015). Germline PTEN, SDHB-D, and KLLN alterations in endometrial cancer patients with Cowden and Cowden-like syndromes: an international, multicenter, prospective study. Cancer.

[57] Kagami M, Nagasaki K, Kosaki R (2017). Temple syndrome: comprehensive molecular and clinical findings in 32 Japanese patients. Genet Med.

[58] Ludwig NG, Radaeli RF, Silva MM (2016). A boy with Prader-Willi syndrome: unmasking precocious puberty during growth hormone replacement therapy. Arch Endocrinol Metab.

[59] Cassidy SB (1997). Prader-Willi syndrome. J Med Genet.

[60] Virdis R, Sigorini M, Laiolo A (2000). Neurofibromatosis type 1 and precocious puberty. J Pediatr Endocrinol Metab.

[61] Henske EP, Jóźwiak S, Kingswood JC, Sampson JR, Thiele EA (2016). Tuberous sclerosis complex. Nat Rev Dis Primers.

[62] Partsch CJ, Japing I, Siebert R, Gosch A, Wessel A, Sippell WG, Pankau R (2002). Central precocious puberty in girls with Williams syndrome. J Pediatr.

[63] Silver HK, Kiyasu W, George J, Deamer WC (1953). Syndrome of congenital hemihypertrophy, shortness of stature, and elevated urinary gonadotropins. Pediatrics.

[64] North KN, Fulton AB, Whiteman DA (1995). Identical twins with Cohen syndrome. Am J Med Genet.

[65] Küry S, Besnard T, Ebstein F (2017). De novo disruption of the proteasome regulatory subunit psmd12 causes a syndromic neurodevelopmental disorder. Am J Hum Genet.

[66] Concolino D, Muzzi G, Pisaturo L, Piccirillo A, Di Natale P, Strisciuglio P (2008). Precocious puberty in Sanfilippo IIIA disease: diagnosis and follow-up of two new cases. Eur J Med Genet.

[67] Jung H, Carmel P, Schwartz MS (1999). Some hypothalamic hamartomas contain transforming growth factor alpha, a puberty-inducing growth factor, but not luteinizing hormone-releasing hormone neurons. J Clin Endocrinol Metab.

[68] Judge DM, Kulin HE, Page R, Santen R, Trapukdi S (1977). Hypothalamic hamartoma: a source of luteinizing-hormone-releasing factor in precocious puberty. N Engl J Med.

[69] van Groeninghen JC, Kiesel L, Winkler D, Zwirner M (1998). Effects of luteinising-hormone-releasing hormone on nervous-system tumours. Lancet.

[70] Soriano-Guillén L, Argente J (2019). Central precocious puberty, functional and tumor-related. Best Pract Res Clin Endocrinol Metab.

[71] Aguirre RS, Eugster EA (2018). Central precocious puberty: from genetics to treatment. Best Pract Res Clin Endocrinol Metab.

[72] Canton AP, Seraphim CE, Brito VN, Latronico AC (2019). Pioneering studies on monogenic central precocious puberty. Arch Endocrinol Metab.

[73] Neocleous V, Fanis P, Toumba M (2021). Pathogenic and low-frequency variants in children with central precocious puberty. Front Endocrinol (Lausanne).

[74] Krstevska-Konstantinova M, Charlier C, Craen M (2001). Sexual precocity after immigration from developing countries to Belgium: evidence of previous exposure to organochlorine pesticides. Hum Reprod.

[75] Eugster EA (2019). Update on precocious puberty in girls. J Pediatr Adolesc Gynecol.

[76] Tanner JM, Goldstein H, Whitehouse RH (1970). Standards for children's height at ages 2-9 years allowing for heights of parents. Arch Dis Child.

[77] Yoon JS, So CH, Lee HS, Lim JS, Hwang JS (2018). Prevalence of pathological brain lesions in girls with central precocious puberty: possible overestimation?. J Korean Med Sci.

[78] Haddad NG, Eugster EA (2019). Peripheral precocious puberty including congenital adrenal hyperplasia: causes, consequences, management and outcomes. Best Pract Res Clin Endocrinol Metab.

[79] Schoelwer M, Eugster EA (2016). Treatment of peripheral precocious puberty. Endocr Dev.

[80] Corica D, Aversa T, Pepe G, De Luca F, Wasniewska M (2018). Peculiarities of precocious puberty in boys and girls with McCune-Albright syndrome. Front Endocrinol (Lausanne).

[81] Carel JC, Eugster EA, Rogol A (2009). Consensus statement on the use of gonadotropin-releasing hormone analogs in children. Pediatrics.

[82] Neely EK, Hintz RL, Wilson DM, Lee PA, Gautier T, Argente J, Stene M (1995127404610). Normal ranges for immunochemiluminometric gonadotropin assays. J Pediatr.

[83] Resende EA, Lara BH, Reis JD, Ferreira BP, Pereira GA, Borges MF (2007). Assessment of basal and gonadotropin-releasing hormone-stimulated gonadotropins by immunochemiluminometric and immunofluorometric assays in normal children. J Clin Endocrinol Metab.

[84] Houk CP, Kunselman AR, Lee PA (2009). Adequacy of a single unstimulated luteinizing hormone level to diagnose central precocious puberty in girls. Pediatrics.

[85] Pasternak Y, Friger M, Loewenthal N, Haim A, Hershkovitz E (2012). The utility of basal serum LH in prediction of central precocious puberty in girls. Eur J Endocrinol.

[86] Lee DS, Ryoo NY, Lee SH, Kim S, Kim JH (2013). Basal luteinizing hormone and follicular stimulating hormone: is it sufficient for the diagnosis of precocious puberty in girls?. Ann Pediatr Endocrinol Metab.

[87] Kaplowitz PB (2020). For premature thelarche and premature adrenarche, the case for waiting before testing. Horm Res Paediatr.

[88] Kaplowitz P, Bloch C (2016). Evaluation and referral of children with signs of early puberty. Pediatrics.

[89] Fuqua JS (2013). Treatment and outcomes of precocious puberty: an update. J Clin Endocrinol Metab.

[90] Sathasivam A, Garibaldi L, Shapiro S, Godbold J, Rapaport R (2010). Leuprolide stimulation testing for the evaluation of early female sexual maturation. Clin Endocrinol (Oxf).

[91] Parent AS, Teilmann G, Juul A, Skakkebaek NE, Toppari J, Bourguignon JP (2003). The timing of normal puberty and the age limits of sexual precocity: variations around the world, secular trends, and changes after migration. Endocr Rev.

[92] Soriano-Guillén L, Argente J (2011). Central precocious puberty: epidemiology, etiology, diagnosis and treatment (Article in Spanish). An Pediatr (Barc).

[93] Potau N, Ibáñez L, Sentis M, Carrascosa A (1999). Sexual dimorphism in the maturation of the pituitary-gonadal axis, assessed by GnRH agonist challenge. Eur J Endocrinol.

[94] Bayer LM (1959). Radiographic atlas of skeletal development of the hand and wrist. Calif Med.

[95] Malina RM, Beunen GP (2002). Assessment of skeletal maturity and prediction of adult height (TW3 method). Am J Hum Biol.

[96] Prokop-Piotrkowska M, Marszałek-Dziuba K, Moszczyńska E, Szalecki M, Jurkiewicz E (2021). Traditional and new methods of bone age assessment - an overview. J Clin Res Pediatr Endocrinol.

[97] Xie Q, Kang Y, Zhang C (2022). The role of kisspeptin in the control of the hypothalamic-pituitary-gonadal axis and reproduction. Front Endocrinol (Lausanne).

[98] Belchetz PE, Plant TM, Nakai Y, Keogh EJ, Knobil E (1978). Hypophysial responses to continuous and intermittent delivery of hypopthalamic gonadotropin-releasing hormone. Science.

[99] Shim YS, Lim KI, Lee HS, Hwang JS (2020). Long-term outcomes after gonadotropin-releasing hormone agonist treatment in boys with central precocious puberty. PLoS One.

[100] Eugster EA (2019). Treatment of central precocious puberty. J Endocr Soc.

[101] Toro CA, Aylwin CF, Lomniczi A (2018). Hypothalamic epigenetics driving female puberty. J Neuroendocrinol.

[102] Carel JC, Lahlou N, Jaramillo O (2002). Treatment of central precocious puberty by subcutaneous injections of leuprorelin 3-month depot (11.25 mg). J Clin Endocrinol Metab.

[103] Johnson SR, Nolan RC, Grant MT, Price GJ, Siafarikas A, Bint L, Choong CS (2012). Sterile abscess formation associated with depot leuprorelin acetate therapy for central precocious puberty. J Paediatr Child Health.

[104] Park J, Kim JH (2017). Change in body mass index and insulin resistance after 1-year treatment with gonadotropin-releasing hormone agonists in girls with central precocious puberty. Ann Pediatr Endocrinol Metab.

[105] Yang WJ, Ko KH, Lee KH, Hwang IT, Oh YJ (2017). The different effects of gonadotropin-releasing hormone agonist therapy on body mass index and growth between normal-weight and overweight girls with central precocious puberty. Ann Pediatr Endocrinol Metab.

[106] Censani M, Feuer A, Orton S, Askin G, Vogiatzi M (2019). Changes in body mass index in children on gonadotropin-releasing hormone agonist therapy with precocious puberty, early puberty or short stature. J Pediatr Endocrinol Metab.

[107] Lee SJ, Yang EM, Seo JY, Kim CJ (2012). Effects of gonadotropin-releasing hormone agonist therapy on body mass index and height in girls with central precocious puberty. Chonnam Med J.

[108] Eugster EA, Clarke W, Kletter GB (2007). Efficacy and safety of histrelin subdermal implant in children with central precocious puberty: a multicenter trial. J Clin Endocrinol Metab.

[109] Paesano PL, Colantoni C, Mora S (2019). Validation of an accurate and noninvasive tool to exclude female precocious puberty: pelvic ultrasound with uterine artery pulsatility index. AJR Am J Roentgenol.

[110] Bangalore Krishna K, Fuqua JS, Rogol AD (2019). Use of gonadotropin-releasing hormone analogs in children: update by an international consortium. Horm Res Paediatr.

[111] Neely EK, Silverman LA, Geffner ME, Danoff TM, Gould E, Thornton PS (2013). Random unstimulated pediatric luteinizing hormone levels are not reliable in the assessment of pubertal suppression during histrelin implant therapy. Int J Pediatr Endocrinol.

[112] Badaru A, Wilson DM, Bachrach LK (2006). Sequential comparisons of one-month and three-month depot leuprolide regimens in central precocious puberty. J Clin Endocrinol Metab.

[113] Acharya SV, Gopal RA, George J, Bandgar TR, Menon PS, Shah NS (2009). Utility of single luteinizing hormone determination 3 h after depot leuprolide in monitoring therapy of gonadotropin-dependent precocious puberty. Pituitary.

[114] Demirbilek H, Alikasifoglu A, Gonc NE, Ozon A, Kandemir N (2012). Assessment of gonadotrophin suppression in girls treated with GnRH analogue for central precocious puberty; validity of single luteinizing hormone measurement after leuprolide acetate injection. Clin Endocrinol (Oxf).

[115] White PC (2018). Update on diagnosis and management of congenital adrenal hyperplasia due to 21-hydroxylase deficiency. Curr Opin Endocrinol Diabetes Obes.

[116] Javaid MK, Boyce A, Appelman-Dijkstra N (2019). Best practice management guidelines for fibrous dysplasia/McCune-Albright syndrome: a consensus statement from the FD/MAS international consortium. Orphanet J Rare Dis.

[117] Zou CC, Liang L, Dong GP, Zhao ZY (2008). Peripheral precocious puberty: a retrospective study for six years in Hangzhou, China. J Paediatr Child Health.

[118] Feuillan P, Calis K, Hill S, Shawker T, Robey PG, Collins MT (2007). Letrozole treatment of precocious puberty in girls with the McCune-Albright syndrome: a pilot study. J Clin Endocrinol Metab.

[119] Papanikolaou A, Michala L (2015). Autonomous ovarian cysts in prepubertal girls. How aggressive should we be? A review of the literature. J Pediatr Adolesc Gynecol.

[120] Agopiantz M, Journeau P, Lebon-Labich B, Sorlin A, Cuny T, Weryha G, Leheup B (2016). McCune-Albright syndrome, natural history and multidisciplinary management in a series of 14 pediatric cases. Ann Endocrinol (Paris).

[121] Leschek EW, Jones J, Barnes KM, Hill SC, Cutler GB Jr (1999). Six-year results of spironolactone and testolactone treatment of familial male-limited precocious puberty with addition of deslorelin after central puberty onset. J Clin Endocrinol Metab.

[122] Almeida MQ, Brito VN, Lins TS (2008). Long-term treatment of familial male-limited precocious puberty (testotoxicosis) with cyproterone acetate or ketoconazole. Clin Endocrinol (Oxf).

[123] Banerjee S, Bajpai A (2023). Precocious puberty. Indian J Pediatr.

